# MoNETA: massive parallel application of biological models navigating through virtual Morris water maze and beyond

**DOI:** 10.1186/1471-2202-12-S1-P310

**Published:** 2011-07-18

**Authors:** Anatoli Gorchetchnikov, Jasmin Leveille, Massimiliano Versace, Heather M Ames, Gennady Livitz, Benjamin Chandler, Ennio Mingolla, Dick Carter, Rick Amerson, Hisham Abdalla, Shakeel M Qureshi, Greg Snider

**Affiliations:** 1 Department of Cognitive and Neural Systems, Boston University, Boston, MA 02215, USA; 2 Hewlett-Packard Laboratories, Palo Alto, CA 94304, USA

## 

The primary goal of a Modular Neural Exploring Traveling Agent (MoNETA) project is to create an autonomous agent capable of object recognition and localization, navigation, and planning in virtual and real environments. Major components of the system perform sensory object recognition, motivation and rewards processing, goal selection, allocentric representation of the world, spatial planning, and motor execution. MoNETA is based on the real time, massively parallel, Cog Ex Machina environment co-developed by Hewlett-Packard Laboratories and the Neuromorphics Lab at Boston University. The agent is tested in virtual environments replicating neurophysiological and psychological experiments with real rats. The currently used environment replicates the Morris water maze [1].

The motivational system represents the internal state of the agent that can be adjusted by sensory inputs. In the Morris water maze, only one drive can be satisfied (a desire to get out of the water) that persists as long as the animat is swimming and sharply decreases as soon as it is fully positioned on the platform. Another drive – curiosity – is constantly active and is never satisfied. It forces the agent to explore unfamiliar parts of the environment. Familiarity with environmental locations provides inhibition to the curiosity drive in a selective manner, so that recently explored locations are less appealing than either unexplored locations or locations that were explored long time ago. The main output of the motivational system is a goal selection map. It is based on competition between goals set by the curiosity system and goals learned by the animat. The goal selection map uses a winner-take-all selection of the most prominent input signal as a winning goal. Because curiosity-driven goals receive weaker inputs than well-learned reward locations, they can only win if there are no prominent inputs corresponding to the learned goals with an active motivational drive.

The spatial planning system is built around a previously developed neural algorithm for goal-directed navigation [2]. The original model provided the desired destination and left it up to the virtual environment to move the animat in this location. In MoNETA the model was extended by a chain of neural populations that convert the allocentric desired destination into an allocentric desired direction and further into a rotational velocity motor command. A second extension of the model deals with the mapping of the environment. The original algorithm included goal and obstacle information into path planning, but this information was provided in the form of allocentric maps where the locations of both the goals and obstacles were received directly from the environment. MoNETA uses these maps, but also creates them from egocentric sensory information through a process of active exploration. Although the current version only uses somatosensory information, visual input will be added in later stages. The system converts egocentric representations to allocentric ones and then learns the mapping of obstacles and goals in the environment. It uses a learning rule that is local to dendrites and does not require any postsynaptic activity.

The complete implementation of MoNETA consists of 75,301 neurons and 1,362,705 synapses.
